# SETDB1, HP1 and SUV39 promote repositioning of 53BP1 to extend resection during homologous recombination in G2 cells

**DOI:** 10.1093/nar/gkv722

**Published:** 2015-07-22

**Authors:** Meryem Alagoz, Yoko Katsuki, Hideaki Ogiwara, Tomoo Ogi, Atsushi Shibata, Andreas Kakarougkas, Penny Jeggo

**Affiliations:** 1 University of Sussex Genome Damage and Stability Centre, East Sussex, BN19RQ, UK; 2 Division of Genome Biology, National Cancer Centre Japan Research Institute, Tokyo, 104-0045, Japan; 3 Department of Molecular Medicine, Atomic Bomb Disease Institute, Nagasaki University, Nagasaki, 852-8523, Japan; 4 Department of Biology, School of Sciences and Engineering, The American University in Cairo, New Cairo, 11835, Egypt

## Abstract

Recent studies have shown that homologous recombination (HR) requires chromatin repression as well as relaxation at DNA double strand breaks (DSBs). HP1 and SUV39H1/2 are repressive factors essential for HR. Here, we identify SETDB1 as an additional compacting factor promoting HR. Depletion of HP1, SUV39, SETDB1 or BRCA1 confer identical phenotypes. The repressive factors, like BRCA1, are dispensable for the initiation of resection but promote the extension step causing diminished RPA or RAD51 foci and HR in irradiated G2 cells. Depletion of the compacting factors does not inhibit BRCA1 recruitment but at 8 h post IR, BRCA1 foci are smaller and aberrantly positioned compared to control cells. BRCA1 promotes 53BP1 repositioning to the periphery of enlarged foci and formation of a devoid core with BRCA1 becoming enlarged and localized internally to 53BP1. Depletion of the compacting factors precludes these changes at irradiation-induced foci. Thus, the repressive factors are required for BRCA1 function in promoting the repositioning of 53BP1 during HR. Additionally, depletion of these repressive factors in undamaged cells causes diminished sister chromatid association at centromeric sequences. We propose a model for how these findings may be functionally linked.

## INTRODUCTION

DNA non-homologous end-joining (NHEJ) and homologous recombination (HR) represent the two major DNA double strand break (DSB) repair pathways in mammalian cells. NHEJ functions throughout the cell cycle whilst HR functions uniquely in late S/G2 phase when a sister chromatid is available as the source of an undamaged template (1). Current models support the notion that NHEJ is exploited as the first option to repair DSBs in G2 phase but if rejoining does not rapidly ensue, then resection is initiated by CtIP and the MRE11/RAD50/NBS1 (MRN) complex, which commits to repair by HR (2–4). Resection is proposed to arise via a two-step process with CtIP and MRE11 endonuclease activity functioning in an initiating event and MRE11 exonuclease activity, exonuclease I and/or DNA2/BLM promoting the extension of resection (3,5–7). siRNA-mediated depletion of CtIP (siRNA CtIP) or treatment with MRE11 endonuclease inhibitors prevent HR but allows DSB rejoining to proceed by NHEJ; in contrast, blocking the extension of resection, precludes the opportunity to utilize NHEJ and confers a DSB repair defect. Following RPA loading onto the ssDNA generated by the initiation of resection, RAD51 subsequently replaces RPA generating nucleoprotein filaments (4). The elongation of resection enhances both RPA recruitment and RAD51 loading. An important further step in HR is the assimilation of the displaced ssDNA onto the undamaged template creating a Holliday junction and D-loop formation, which can be considered to represent a step of synapsis. Finally, post synapsis events include fill-in by polymerases, branch migration and Holliday junction resolution.

BRCA1 also plays a critical role in the extension step of resection (8,9). Current models propose that 53BP1, a damage response mediator protein, which is recruited to DSBs in a choreographed manner at irradiation induced foci (IRIF), functions to promote NHEJ by restricting resection. Recent studies have, in fact, suggested that phosphorylation of 53BP1 leads to the recruitment of RIF1, which represents the effector factor restricting resection (10–12). Whilst depletion of BRCA1 causes a defect in DSB repair and impeded resection, co-depletion of BRCA1 and CtIP allows DSB repair by NHEJ without resection suggesting that BRCA1 functions downstream of CtIP to promote the extension of resection. Intriguingly, loss of BRCA1 and 53BP1 or BRCA1 and RIF1 allows resection and HR to proceed suggesting that BRCA1 functions to relieve the barrier posed by 53BP1/RIF1. Consistent with this, two studies have shown that 53BP1 foci expand in G2 phase to create a devoid core via a BRCA1-dependent process. This repositioning of 53BP1 is required for the extension step of HR.

Chromatin changes in the DSB vicinity are critical to the DNA damage response (DDR). Whilst chromatin relaxation promoted by ubiquitination and DDR protein recruitment has been well described, more recent studies have provided evidence that compacting factors are also recruited at least transiently at DSB sites and that they are required for the completion of HR (13–19). Such factors have now been shown to include HP1, SUV39H1 and KAP1. However, their function during HR has not been well examined. Here, we identify the histone methyltransferase, SETDB1 (also called ESET/KMT1E), which also contributes to chromatin compaction via an ability to methylate histone H3K9, as a further compacting protein being recruited to DNA DSBs and being essential for the completion of HR (20–24). We then define the position at which they function during HR. HP1, SUV39 and SETDB1, like BRCA1, all function in the extension step of resection, downstream of the initiation step. They are dispensable for the initial recruitment of BRCA1 but their depletion impairs the correct positioning of BRCA1 relative to 53BP1 and importantly prevents the two-fold enlargement of 53BP1 at IRIF, which takes place in G2 cells during HR (25,26). Interestingly, depletion of HP1, SUV39 or SETDB1 in undamaged cells reduces sister chromatin association at centromeric regions. These findings define SETDB1 as being a further component required for HR, they show that the compacting factors have a downstream role in HR and raise the possibility that they promote synapsis or engagement of the damaged strand with the undamaged strand.

## MATERIALS AND METHODS

### Cell culture and irradiation

Human hTERT-immortalized fibroblasts (1BR3 hTERT) and A549 cells were cultured in Minimum Essential Medium (MEM), U2OS DR-GFP cells were cultured in Dulbecco's Modification of Eagles Medium (DMEM) supplemented with 10% fetal calf serum (PAA laboratories), 2mM L-glutamine (Gibco), 100 unit/ml penicillin (Gibco) and 100 unit/ml streptomycin (Gibco) at 37°C in a humidified 95% air and 5% CO_2_ atmosphere. Cells were irradiated by exposure to a Cs source. U2OS DR126 green fluorescent protein (GFP) cells were kindly provided by Dr M. Jasin (Gillingham, UK). A total of 3 μM aphidicolin (APH) was added to cells 30 min prior to ionizing radiation (IR) to prevent S-phase cells from progressing into G2 (2,27). Mitotic cells were excluded by their characteristic chromatin condensation and S phase cells by pan-nuclear γH2AX staining. G2 cells were identified using CENPF staining (a G2 marker).

### Small interfering RNA knockdown and plasmid transfection

Small interfering RNA (siRNA) mediated gene knockdown was performed using Hyperfect transfection reagent (Qiagen, Hilden, Germany) following the manufacturer's instructions. A total of 20 pM siRNA were transfected per 2 × 10^4^ cells. Cells were grown for 72 h before IR. SMARTpool siRNA oligonucleotides from Dharmacon were used for Control, HP1α, HP1β, HP1γ, CtIP, EXO1 and BLM. SUV39H1 (5′-ACCUCUUUGACCUGGACUAT-3′) and SUV39H2 (5′-GAAGCUACCUUUGGUUGUUTT-3′) siRNA oligonucleotides were obtained from Thermo Scientific. SETDB1A (5′-GGAACUGGAGAAGAUGGAUUGUGUA-3′) and SETDB1B (5′-CCGTGAAGCTATGGCTGCCTTAAGA-3′) were from Dharmacon. BRCA1 (5′-GGAACCUGUCUCCACAAAG-3′) siRNAs were synthesized by Invitrogene (UK). Unless otherwise stated, combined HP1α/β siRNA oligonucleotides were used. Combined SETDB1A/B and combined SUV39H1/H2 were used in all experiments.

The pCBASceI (from Dr M Jasin) plasmid was transfected using GeneJuice transfection reagent (Novagen, Germany) per 2.5 × 105 cells at 24 h post siRNA transfection.

### Immunofluorescence and foci analysis

Cells grown on glass slides were fixed with paraformaldehyde (PFA) and permeabilized with 0.2% Triton X-100. For RPA, RAD51 and BRCA1 staining, cells were pre-extracted with 0.2% Triton X-100 in phosphate buffered saline (PBS) before fixation. Cells were incubated with primary antibody and followed by several washes with PBS before incubation with secondary antibodies. Slides were then mounted by Vectashield and analysed using a Nikon-e400 microscope. Foci scoring were performed as described previously (2). Images were taken by using an Applied Precision^®^ Delta Vision^®^ RT Olympus IX70 deconvolution microscope and softWoRx^®^ Suite software. The percentage of overlapping foci was assessed using the Pearson coefficient of correlation, which monitors overlap on a pixel-by-pixel basis (full overlap is 1.0). Overlapping analysis was undertaken using softWoRx^®^ Suite software. Z-stack imaging and 3D modelling and foci volume quantification were done as described previously (26). Foci scoring were performed as described previously (2). Mean values represent 15–20 cells in each of three experiments for each condition. The fluorescence intensity was measured along a line drawn through the centre of foci at different times after IR. Line intensity plots were performed using the softWoRx^®^ suite software.

### Immunoblotting

For Immunoblotting, cells were lysed in high salt lysis buffer, IPLB (50 mM Tris–HCl, 500 mM NaCl, 2 mM EDTA, 2 mM EGTA, 25 mM NaF, 25 mM β-glycerol phosphate, 0.1 mM NaOrthovanadate, 0.2% Triton X-100, 0.3% NP-40, 10 U/ml of Benzonase nuclease, plus protease inhibitor cocktail (Roche, Basel, Switzerland) at 4°C. Total proteins were separated by 10% sodium dodecyl sulphate-polyacrylamide gel electrophoresis and transferred to nitrocellulose membranes. Membranes were blocked in 6% non-fat dry milk and incubated with primary antibodies followed by incubation with horse-radish peroxidase (HRP) conjugated secondary antibodies and developed with Enhanced Chemiluminescence (Western lightening Plus-ECL, Perkin Elmer).

### Antibodies for immunofluorescence and immunoblotting

The primary antibodies were: γH2AX (1:800, Upstate Technology, USA), 53BP1 (1:1000, Bethyl, Cambridge, UK), RPA (1:100 for IF, Calbiochem, USA), RAD51 (1:200 for IF and 1:500 for IB, Santa Cruz Biotechnology, USA), BRCA1 (1:100 for IF and 1:50 for IB, Santa Cruz), HP1(α,β,γ) (1:1000 for IB, Santa Cruz). HP1α (1:500 for IB, Santa Cruz), RIF-1 (1:1000 Bethyl, Cambridge, UK), SUV39H1/2 (1:300 for IB, Santa Cruz), SETDB1 (1:100 for IF, Abcam, UK, 1:1000 for IB, Cell Signalling Technology (CST), EXO1 and BLM (1:500, Santa Cruz), H3 (1:3000 Abcam, UK), H3K9 (1:3000 from Bethyl, Cambridge, UK), β-tubulin (1:3000, Sigma Aldrich, UK), HA tag (1:1000, Abcam) and mouse monoclonal anti-Actin (1:3000 from Sigma Aldrich, UK). The secondary antibodies were FITC (1:200, Sigma Aldrich, UK), Cy3 (1:200, Sigma Aldrich, UK) and Alexa Fluor 488 (1:400, Invitrogen, USA). Horseradish peroxidase (HRP) conjugated secondary antibodies were from Dako, UK.

### Premature chromosome condensation (PCC) breakage

Premature chromosome condensation (PCC) analysis was carried out as described previously (28). Briefly, 1BR3 hTERT cells were irradiated with 2 Gy IR after adding 3 μM APH to prevent S-phase cells from progressing into G2 (2,27). Cells were treated with 50 ng/ml Calyculin-A for 30 min to induce PCC in G2-phase cells. Cells were then harvested at the indicated time points and processed for chromosome break analysis. Chromosome breaks were scored in 100 chromosomes.

### HR and NHEJ mediated DR-GFP reporter assay using fluorescene-activated cell sorting

U2OS DR-GFP and H1299 dA3–1 cells were used for HR and NHEJ assays, respectively (3,29,30). Cells were transfected with HA tagged *I-SceI* plasmid (pCBA*SceI*) or pEGFP-C3 empty vector 24 h after siRNA transfection. Forty-hours later, cells were trypsinized, resuspended in PBS and transferred to ice in the dark. GFP expression was analysed using a fluorescence activated cell sorter (FACSCanto, BD Biosciences) with FACS Diva software. The GFP expressing population was normalized by the percentage of the population expressing enhanced green fluorescent protein (EGFP) as well as the magnitude of the S/G2 phase population. For analysis of cell cycle distribution, cells were washed and resuspended in PBS containing propidium iodide with RNase A (Sigma-Aldrich). Western blot analysis was performed to assess *I-SceI* protein levels after each siRNA treatment. Expression of HA-tagged *I-SceI* was assessed by staining with HA tag antibody (31).

### Laser micro-irradiation

To generate localized damage in cellular DNA with a diode laser beam, cells were grown on glass bottom dishes (IWAKI, Japan) and pre-sensitized with 15 μM 5-bromo-2-deoxyuridine (BrdU, Sigma-Aldrich, USA) in DMEM for 48 h at 37°C. Laser micro-irradiation was performed using a LSM700 laser scan 209 confocal microscope (Carl Zeiss) equipped with a 405 nm LED laser diode (5 mW). The laser was focused through a 40× Plan-Apochromat/1.3 oil objective and operated at 100% power output with 10 iterations. The pixel dwell time to expose cells to the laser beam was 50 μs. The regions in cellular DNA were irradiated in 10–15 min. After laser irradiation, cells were incubated for 20 min at 37°C and then fixed as described for IF analysis. All laser-track images were acquired on an Axio Observer inverted microscope (Carl Zeiss) with AxioVision software.

### Laser micro-irradiation using live cell imaging

U2OS cells were seeded onto 35 mm glass-bottom dishes (MatTek) and transfected with pEGFP-SETDB1 constructs using Gene Juice transfection reagent. Cells were grown for 48 h and, when used, 10 μM ATMi (Tocris Bioscience) added 1 h prior to the exposure to laser microirradiation. Cells were incubated with 10 μg/ml Hoechst 34580 for 30 min at 37°C before exposure. The Intelligent Imaging Innovations spinning disk confocal microscopy with a Yokogawa CSU-X1 on an Olympus IX-71 was used for imaging. EGFP positive cells were irradiated with a 50 mW, 405 nm ultraviolet laser and channelled through a 60× objective. The UV laser was focused to an area of ∼12 × 0.1μm through the cell nuclei, and images were captured at 10 s intervals following laser damage for a total time of 210. Signal intensity was quantified along the laser path using Slidebook 6 software. As controls, cells were exposed to laser microirradiation without Hoescht treatment and no signal was obtained in all cases.

### Fluorescence in situ hybridization (FISH) assay

Cells were transfected with siRNA oligonucleotides and subjected to immunofluorescence analysis to identify G2 cells using CENPF antibody (27). Following immunoflourescence, cells were fixed with 3% PFA and permeabilized by HCL/Triton X-100. DNA was denatured with 50% formamide and hybridized with the probe as described by the manufacturer. DiGeorge II (10p14) probe contains a probe specific to satellite DNA located at the centromeric region on Chromosome 10 (labelled in green) (D10Z1). A minimum of 70 cells were imaged using an Applied Precision^®^ Delta Vision^®^ RT Olympus IX70 deconvolution microscope and data analysed using softWoRx^®^ Suite software.

### Statistical analysis

All data were derived from three or four independent experiments unless stated. Box plot was created by SigmaPlot 12.0. Statistical significance was determined using Mann–Whitney U test or Student's two-tailed *t*-test by SigmaPlot 12.0. Significance was indicated as *P < 0.05, **P < 0.01, ***P < 0.001.

## RESULTS

### HP1, SUV39 and SETDB1 are dispensable for NHEJ but are required for HR

**Figure 1.**
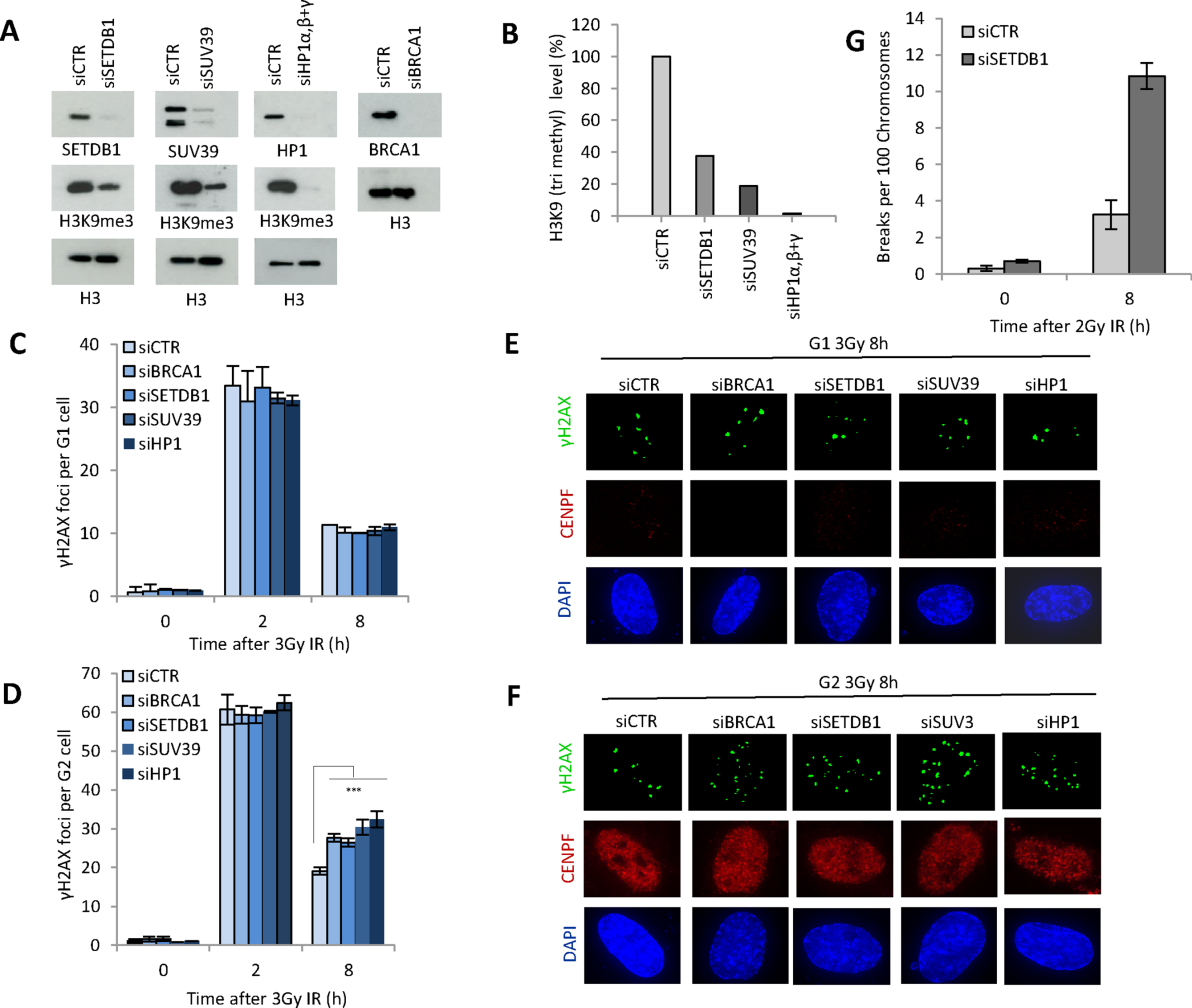
SETDB1, SUV39 and HP1 are dispensable for DSB repair in G1 phase but essential in G2. (**A**) 1BR3 hTERT cells were transfected with siRNAs targeting control (CTR), SETDB1, SUV39, HP1α/β and γ and BRCA1. Knockdown of each component was verified by immunoblotting and H3K9me3 levels assessed in whole cell extracts. (**B**) Quantification of the reduction in H3K9me3 levels. Note that in panels A and B we used combined oligonucleotides for HP1α/β and γ. (**C** and **D**) Following siRNA mediated knockdown as indicated, 1BR3 hTERT cells were irradiated with 3 Gy and γH2AX foci enumerated at the indicated times in (C) G1 (CENPF^−^) and (D) G2 (CENP^+^) cells. (**E** and **F**) Representative images from experiments (C and D). Staining for γH2AX, CENPF and DAPI is as indicated. Asterisks denote statistically significant differences (*P* < 0.05); *t*-test). Results represent the mean ± S.E.M of 3 experiments. (**G**) Chromosomal breaks were determined by premature chromosome condensation (PCC) breakage analysis. 1BR3 hTERT cells were subjected to siRNA mediated knockdown of control (CTR) and SETDB1 and irradiated with 2 Gy IR. Chromosomal breaks were assessed at 8 h post IR. Data are represent the mean ± S.E.M of three experiments.

To facilitate an assessment of compacting factors on HR, we first established siRNA mediated depletion conditions that impair chromatin compaction without impeding cell cycle progression. Unless otherwise stated, we examined combined depletion of HP1α and β (called HP1), combined depletion of SUV39H1/H2 (called SUV39), components previously described as influencing HR, and combined depletion of the histone methyl transferase, SETDB1A/B (called SETDB1), which has not been previously examined (15,19). Using the optimally chosen siRNA-mediated depletion conditions, we observed reduced levels of each factor and diminished H3K9me3 levels without any substantial reduction in the magnitude of G2 cells (Figure 1A, B and Supplementary Figure S1A). To assess the influence on DSB repair, we enumerated γH2AX foci following exposure to IR and depletion of each factor (siRNA HP1, SUV39 or SETDB1) (Figure 1C). siRNA BRCA1 was included as a control to assess the contribution of HR to DSB repair. To monitor DSB repair in cells irradiated and maintained in G1 or G2 phase, we treated cells with APH, which inhibits the replicative polymerases, to identify S phase cells and prevent their progression into G2 during analysis. G2 cells were identified using centromeric protein F (CENPF). Extensive control experiments have shown that APH does not induce DNA damage in G2 cells nor impede DSB repair (2,27). We have previously shown that DSBs are repaired with two component kinetics in G1 and G2 phase, and that the slow process in G2 represents HR (2,27). siRNA HP1, SUV39 or SETDB1 did not affect DSB repair at 2 or 8 h post IR in G1 phase (Figure 1C). Since most DSB repair in G1 occurs by c-NHEJ, we conclude that NHEJ is unaffected by depletion of these proteins. In contrast, whilst DSB repair was normal at 2 h post IR in CENPF^+^ G2 cells, a subtle but statistically significant and reproducible DSB repair defect was observed at 8 h. This defect was identical to that observed following siRNA BRCA1 (Figure 1D). This represents the characterized defect observed following loss of additional HR proteins, either by siRNA-mediated depletion or in mutant mouse embryo fibroblasts (MEFs) (2,27). This represents 15–20% of induced DSBs and is consistent with the notion that NHEJ repairs the majority of DSBs in G2 with fast kinetics whilst HR repairs a subset of DSBs with slow kinetics. To substantiate that depletion of SETDB1 confers a DSB repair defect using a procedure that does not rely on chromatin changes, we also examined chromosome breaks following treatment with calyculin A, which causes premature chromosome condensation of G2 cells (28). We observed elevated chromosome breaks at 8 h consistent with the DSB repair defect revealed by γ-H2AX analysis (Figure 1G). These findings provide suggestive evidence that SETDB1 is required for HR in G2 phase and supports studies reporting roles for HP1 and SUV39 in HR (15,19).

**Figure 2.**
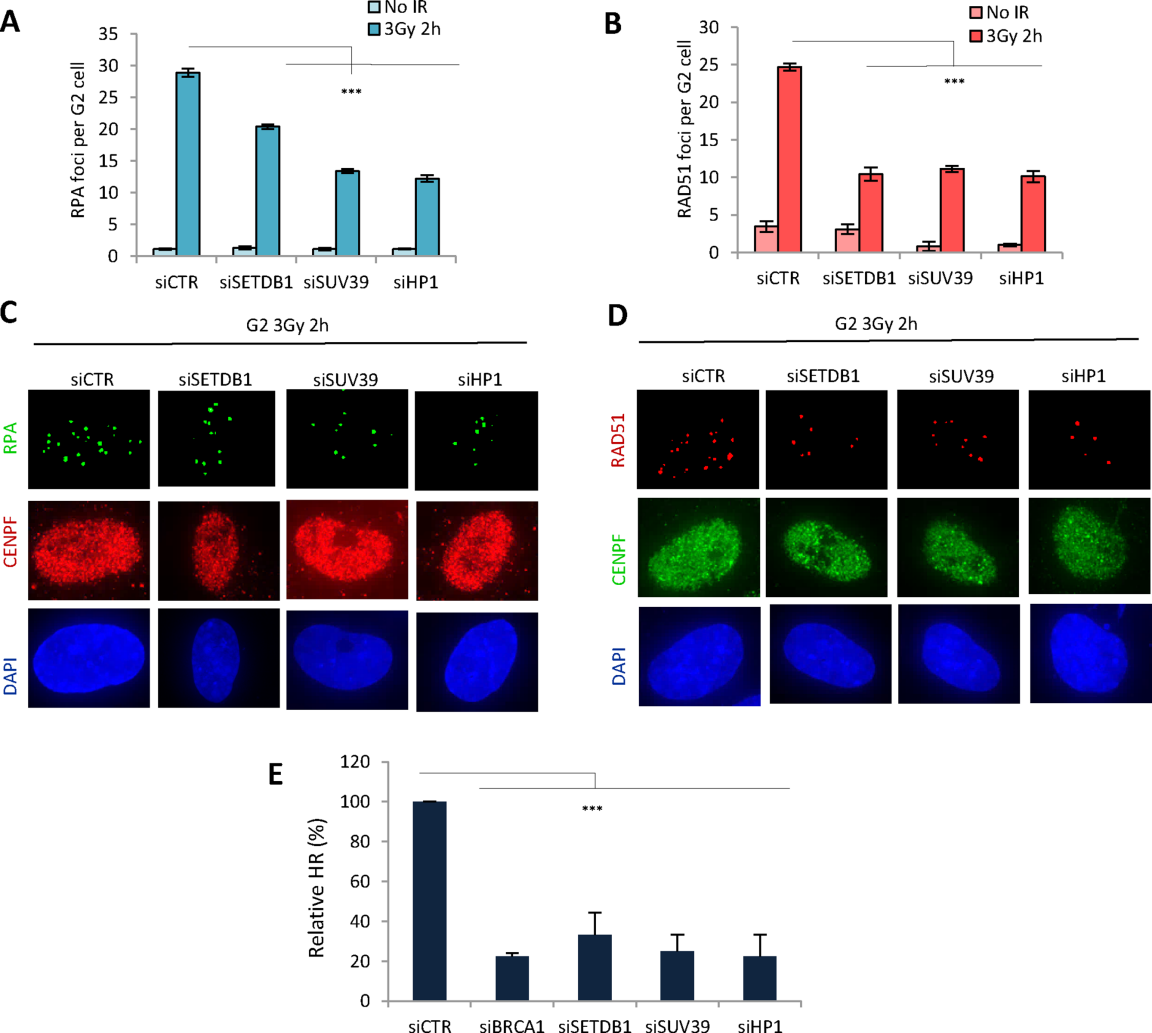
SETDB1, SUV39 and HP1 are required for the formation of RPA and RAD51 foci and for HR. (**A** and **B**) 1BR3 hTERT cells were transfected with siRNAs targeting control (CTR), SETDB1, SUV39, HP1 and BRCA1, irradiated with 3 Gy and 2 h post IR, (A) RPA and (B) RAD51 foci were quantified. RPA and RAD51 analysis at additional time points is shown in Supplementary Figure S1. Asterisks denote statistically significant differences between control (CTR) and SETDB1, SUV39, HP1 knockdown cells. (*P* < 0.001;*t*-test). (**C** and **D**) Representative images from the above experiments: (C) RPA and CENPF (D) RAD51 and CENPF as indicated. **E**. U2OS DR-GFP cells were transfected with siRNAs targeting control (CTR), BRCA1, SETDB1, SUV39 or HP1 and 24 h later were re-transfected with pCBA*SceI*. A GFP signal is generated following intrachromosomal gene conversion following I-*SceI-*induced DSB formation. The percentage of GFP^+^ cells was measured by FACS 48 h after transfection with *I-Sce1*. GFP^+^ cells were normalized to the percentage of EGFP positive cells and the level of the S/G2 population. All samples had a similar fraction of G2 phase cells and expression of *I-Sce1* was shown to be similar by Western blotting using an HA-tag on *I-Sce1* (Supplementary Figure S2A, B). Asterisks denote statistically significant differences (*P*<0.001; *t*-test). Results represent the mean ± S.E.M of 3 experiments.

To substantiate that HR is impaired following depletion of the compacting proteins, we also examined the formation of RPA and RAD51 foci in G2 cells at 2 and 8 h post IR and observed an approximately two-fold defect in both end points (Figure 2A, B, Supplementary Figure S1B and C). This is similar to the typical phenotype observed following siRNA BRCA1 (2). Finally, to consolidate an HR defect, we employed a cell line harbouring an integrated construct that allows HR to be monitored following the introduction of *I-Sce1* induced DSBs (DR-GFP) (Figure 2E) (29). Consistent with the findings above, siRNA HP1, SUV39 or SETDB1, conferred a similar HR defect to that observed following siRNA BRCA1. The cell cycle distribution analysed by FACS was not impacted by the siRNA treatments and *I-Sce1* was expressed at a similar level in all cells (Supplementary Figure S2B and F). Since this represents the first report that siRNA SETDB1 impedes HR, we substantiated the finding using two distinct siRNA SETDB1 oligonucleotides (Supplementary Figure S2C and SD) (the data in Figure 2E and all subsequent analysis used the combined oligonucleotides). Finally, using a similar assay to monitor NHEJ, we observed a normal frequency following siSETDB1, HP1 or SUV39 consistent with the notion that HR is specifically affected (Supplementary Figure S2E and F). Thus, we demonstrate that SETDB1 is dispensable for NHEJ but identify it as an additional factor required for HR.

### HP1, SUV39 and SETDB1 function downstream of the initiation of resection

**Figure 3.**
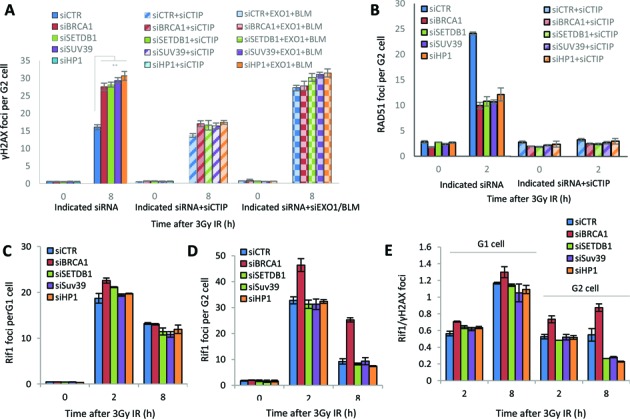
SETDB1, HP1 and SUV39 function downstream of the initiation of resection. (**A**) Following transfection of 1BR3 hTERT cells with siRNAs targeting control (CTR), SETDB1, SUV39, HP1 with or without co-depletion with CtIP or EXO1/BLM, cells were irradiated with 3 Gy and γH2AX foci enumerated at the times indicated. Asterisks denote statistically significant differences (*P<*0.01; *t*-test). Knockdown of EXO/BLM is shown in Supplementary Figure S3A. (**B**) Cells treated as above were enumerated for RAD51 foci at 2 h post IR. (**C** and **D**) Following siRNA mediated knockdown as in panel A, cells were exposed to 3 Gy IR and stained with RIF-1 antibodies at the indicated times. RIF1 foci were counted in (C) G1 and (D) G2 cells. (**E**) The ratio of RIF1/γH2AX foci in G1 versus G2 cells at 2 and 8 h post 3 Gy IR. Data are the mean ± S.E.M of three experiments.

We next sought to dissect the stage during HR when the compacting proteins function. The findings above suggest that their depletion allows some but not extensive resection, a phenotype also observed following BRCA1 depletion. Recent studies have demonstrated that resection occurs via two steps; an initiation step involving CtIP/MRN, which ensures a commitment to HR by preventing the usage of NHEJ, and an elongation stage, which involves EXOI or BLM/DNA2 (3,5–7). The findings above suggest that the compacting proteins might promote the elongation of resection. We previously observed that inhibition of the initiation of resection by siRNA CtIP enabled DSB repair to ensue by NHEJ (2,3). Thus, combined siRNA CtIP + BRCA1 or CtIP + BRCA2 relieves the DSB repair defect conferred by siRNA BRCA1 or BRCA2 without relieving the defect in resection or RAD51 loading (26). We, therefore, carried out combined siRNA CtIP + HP1, SUV39 or SETDB1, and examined DSB repair at 8 h in G2 phase cells, when the contribution of HR to DSB repair can be optimally observed. Consistent with the findings in Figure 1C, we observed a defect in the repair of a subset of DSBs following siRNA HP1, SUV39 or SETDB1 (Figure 3A). Strikingly, this repair defect was relieved when siRNA CtIP was combined with depletion of any of the compacting factors, similar to the findings with siRNA CtIP + BRCA1. Despite efficient DSB repair, combined siRNA CtIP + HP1, SUV39 or SETDB1 further reduced the level of RAD51 foci formation substantiating that DSB repair does not ensue by HR (Figure 3B). These findings consolidate the notion that siRNA CtIP precludes the initiation of resection but allows DSB repair to ensue by NHEJ in G2 phase. EXO1/BLM function downstream of CtIP to extend the resected region (3). We additionally examined whether siRNA EXO1/BLM could influence the repair defect conferred by siRNA HP1, SUV39 or SETDB1 (Figure 3A and Supplementary Figure S3A). Consistent with the notion that EXO1/BLM function downstream of CtIP, we observed a repair defect following siRNA EXO1/BLM and the magnitude of the defect was not changed following combined siRNA EXO1/BLM + HP1 /SUV39 or SETDB1, strongly suggesting the compacting factors function in an epistatic manner to promote the elongation of resection following its initiation. Thus, we conclude that the three chromatin compacting factors are dispensable for the initiation of resection but function to promote the extension step.

RIF1 has been proposed as a factor inhibiting resection, being released from DSBs in G2 phase as HR ensues (10–12). To assess the impact of the compacting factors on RIF1, we examined RIF1 foci formation at times after IR in G1 and G2 cells. Consistent with previous findings, siRNA BRCA1 resulted in elevated RIF1 foci compared to that in control cells most strikingly in G2 phase cells (10–12). Although RIF1 was not present at all γH2AX foci at 2 h in G1 or G2 cells, there was a 1:1 relationship between RIF1 and γH2AX foci by 8 h in control G1 but not G2 cells (Figure 3E). Following BRCA1 depletion, at 8 h in G2 phase the ratio of γH2AX: RIF1 foci was close to 1. In contrast, siRNA of the compacting factors did not affect the level of RIF1 foci compared to control cells in either G1 or G2 (Figure 3C and D). Since there are more DSBs remaining at 8 h in G2 phase cells following siRNA HP1, SUV39 or SETDB1 (Figure 3A), the number of RIF1 foci/γH2AX foci was slightly lower than in control cells although the basis for this is unclear (Figure 3E). Thus, we conclude that depletion of the compacting factors exerts a distinct impact to siRNA BRCA1 and that the defect in resection does not correlate with an inability to remove a barrier caused by RIF1.

### HP1, SUV39 and SETDB1 are dispensable for BRCA1 recruitment but are required for the enlargement of BRCA1 foci in G2

**Figure 4.**
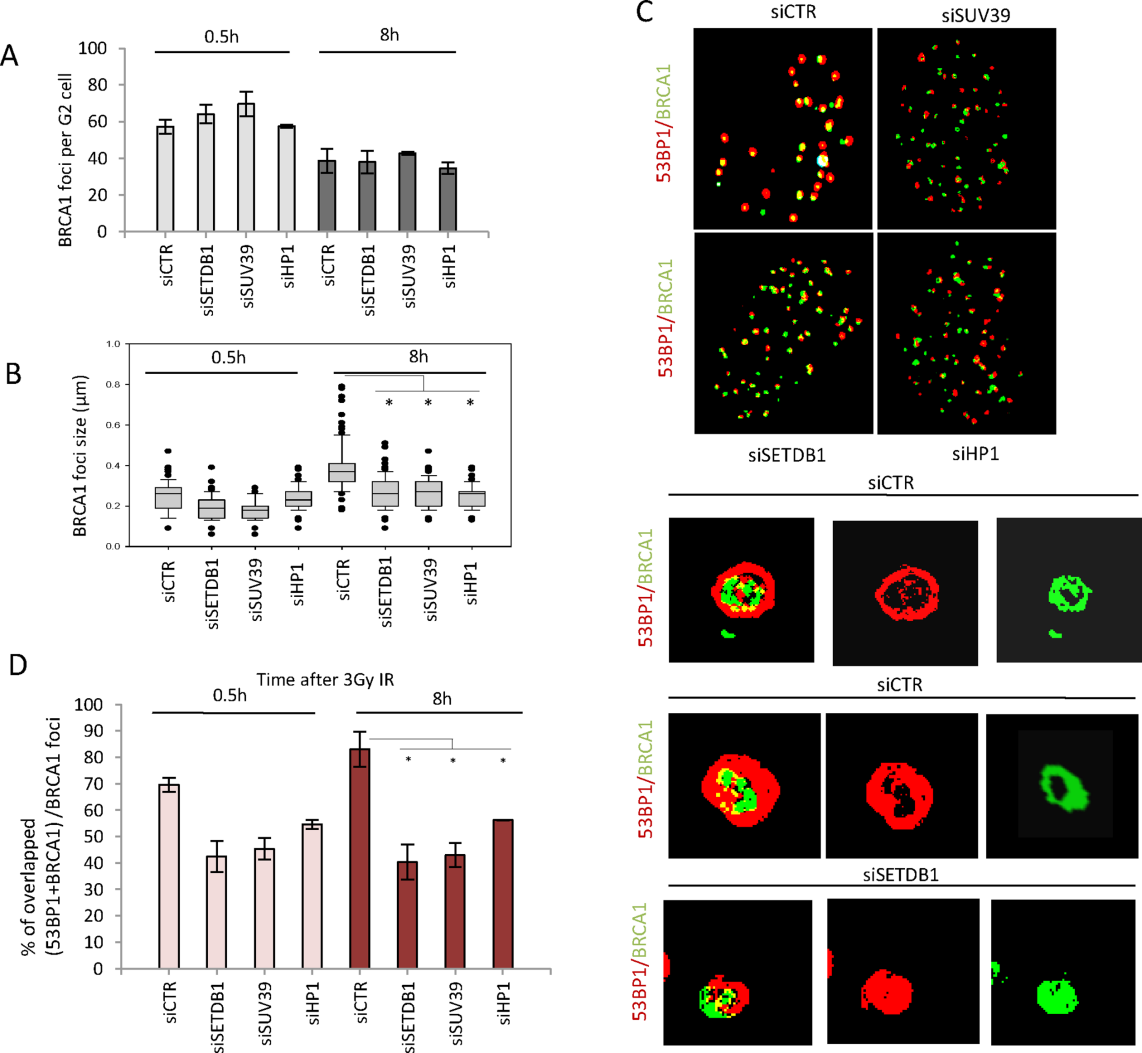
SETDB1, SUV39 and HP1 are dispensable for BRCA1 recruitment but required for its enlargement and localization within IRIF. (**A**) BRCA1 foci were enumerated in 1BR3 hTERT cells at 0.5 and 8 h in G2 cells post 3 Gy IR following the indicated siRNA-mediated knockdown. Note that Delta Vision microscopy was used for this analysis in contrast to Figure 1. The number of foci (γH2AX and BRCA1) is elevated when enumerated using Delta Vision microscopy due to the better delivery of the light source to the objective lens and enhanced contrast. Thus, foci numbers here do not correlate with those in Figure 1 although the relative impact of siRNA remains consistent. (**B**) BRCA1 foci size was analysed in G2 cells after 3 Gy IR at the indicated time points using softWoRx^®^ suite software. Data are the mean ± S.E.M of three experiments. Asterisks denote statistically significant differences (*P* < 0.05; *t*-test). (**C**) 1BR3 hTERT cells were transfected with siRNAs targeting control (CTR) or SETDB1, SUV39, HP1, 48 h later cells were irradiated with 3 Gy and co-stained with BRCA1 and 53BP1 antibody at 8 h post IR. Images were taken by Delta Vision microscopy. In the bottom panel, representative 3D images were generated by the softWoRx^®^ suite software. Panels shows representative images of 53BP1 and/or BRCA1 at 8 h post IR. Top two lines of the bottom panel show two images after siCTR and the bottom row after siRNA SETDB1 (siSETDB1). Images highlight lack of overlap of 53BP1 and BRCA1 foci after knockdown of SETDB1 and the lack of a devoid core. Knockdown of the other compacting factors gave images similar to those after siRNA SETDB1. Note that the top images show a 2D representation and not all foci are therefore observed. (**D**) The overlap of BRCA1 and 53BP1 foci was quantified in deconvolved Delta Vision 2D images by the softWoRx^®^ suite software. The result indicates percentage of overlapping foci between (BRCA1+ 53BP1) relative to total BRCA1 foci. The Pearson coefficient of correlation monitors how closely two intensities overlap on a pixel-by-pixel basis (full overlapping is 1.0). Asterisks indicate statistical difference (*P* < 0.05; *t*-test).

The findings above reveal that depletion of HP1, SUV39 or SETDB1 confers a phenotype similar to that conferred by siRNA BRCA1. This raised the possibility that the compacting factors are required for BRCA1 recruitment. To examine this, we enumerated BRCA1 foci in G2 phase cells at 0.5 and 8 h post 3 Gy IR. The number of BRCA1 foci was reduced at 8 versus 0.5 h, but was not significantly affected by depletion of the compacting factors (Figure 4A). However, given that the number of γH2AX foci was elevated at 8 h following depletion of HP1, SUV39 or SETDB1, the number of γH2AX foci with co-localized BRCA1 foci was actually reduced (data not shown). Notwithstanding this, it is evident that BRCA1 is recruited normally when the compacting factors are depleted.

During this analysis, we observed that BRCA1 foci were consistently smaller at 8 h following depletion of HP1, SUV39 or SETDB1. We exploited Z-stacked immunofluorescence imaging and 3D reconstruction to quantify the size of BRCA1 foci and observed a significant increase in BRCA1 foci size by 8 h in control G2 cells, which did not occur following depletion of the compacting factors (Figure 4B). This increase in BRCA1 foci size during the progression of HR in G2 phase cells is consistent with our previous analysis (25,26). Thus, the reduction in numbers of BRCA1:γH2AX foci numbers discussed above may be a consequence of inefficient estimation of BRCA1 foci numbers due to their small size.

Collectively, these findings provide preliminary evidence that the compacting proteins are dispensable for the initial recruitment of BRCA1 but impede the normal enlargement of BRCA1 foci.

During this analysis, we observed that the centre of BRCA1 and 53BP1 foci did not appear to precisely co-localize as they did in control cells (see Figure 4C for representative images). Using Z-stacked immunofluorescence imaging and 3D reconstruction, we examined the nature of damage response foci at 8 h post IR and in control cells and observed that by 8 h post IR, a devoid core within 53BP1 foci was evident in the majority of foci (Figure 4C). Although slightly less evident, a devoid core was also observed within some of the BRCA1 foci and BRCA1 was located internally to 53BP1, which is consistent with our own and previous work examining the structure of damaged induced foci (Figure 4C) (25,26). Following depletion of any of the three compacting factors, a devoid core was not evident in any foci, and it appeared that 53BP1 and BRCA1 were frequently offset (Figure 4C). We used Delta Vision image analysis software to quantify the percentage of foci where 53BP1 and BRCA1 were overlapping (and hence by deduction were offset). The software assessed foci as overlapping when a pre-determined level of overlap was observed, which did not represent precise localization of BRCA1 in the centre of 53BP1 as shown in control cells in Figure 4C. Thus, our estimate of foci that do not overlap likely represents an underestimation of the aberrant relative position of BRCA1 versus 53BP1 after siRNA of the compacting proteins. Since the number of BRCA1 foci was less than that of 53BP1, we calculated the ratio of BRCA1 and 53BP1 foci assessed by the software to be ‘overlapping’ divided by the total number of BRCA1 foci (Figure 4D). This analysis clearly revealed that the relative positioning of BRCA1 and 53BP1 was changed by depletion of the compacting proteins. This was evident at 0.5 h post IR but became more evident by 8 h when, in control cells BRCA1 and 53BP1 foci have undergone enlargement.

Since, in control cells 53BP1, BRCA1 and γH2AX form concentric foci, albeit of different diameters and with devoid cores of different diameters, we aimed to assess whether 53BP1 or BRCA1 overlapped with γH2AX following siRNA SETDB. We used the same procedure described above, namely assessing the percentage of overlapping BRCA1 or 53BP1 foci with γH2AX foci relative to the total number of γH2AX foci. With control cells, we observed a high level of overlap (>80%) for 53BP1 and γH2AX at 0.5 and 8 h and for BRCA1 and γHAX at 8 h (Supplementary Figure S3B and C). BRCA1 and γH2AX overlap at 0.5 h was slightly lower since BRCA1 foci have not completely formed at this early time point (Supplementary Figure S3C). This demonstrates that despite the presence of the devoid cores and different diameters of the foci, there is high ‘overlap’ assessed by this procedure, most likely due to limitation in resolution. Following siSETDB1, however, the overlap for 53BP1 and γH2AX was substantially lower than observed in controls cells whilst being similar for BRCA1 and γH2AX (Supplementary Figure S3B and C). This strongly suggests that 53BP1 is the DDR protein that becomes incorrectly positioned at the IRIF in the absence of the compacting factors. The underlying basis is not fully understood but suggests that as BRCA1 foci enlarge in G2 phase, 53BP1 becomes inappropriately positioned when chromatin compaction is aberrant.

### HP1, SUV39 and SETDB1 are required for the repositioning of 53BP1 to the periphery of enlarged foci during HR

**Figure 5.**
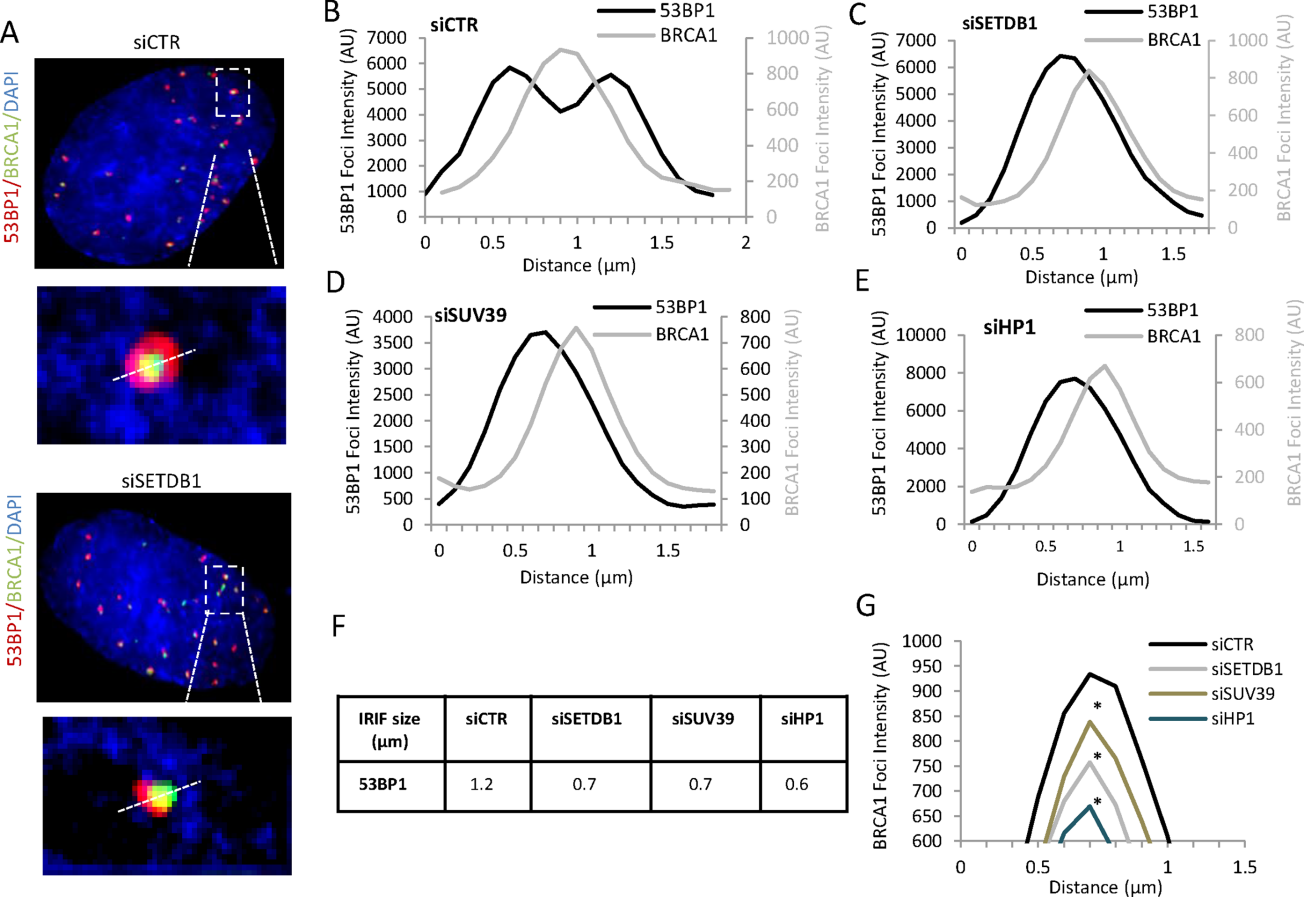
SETDB1, SUV39 and HP1 are required for the repositioning of 53BP1 to the periphery of IRIF during HR. (**A**) Representative images of 53BP1 (red) and BRCA1 (green) foci used to generate the analysis in panels A–D. This figure shows how a line is drawn through the foci. Following siRNA control, 53BP1 entirely surrounds BRCA1 whereas following siRNA SETDB1 (siSETDB1) the two foci are off set. (**B**) A549 cells were depleted with siRNAs targeting control (CTR), SETDB1, SUV39, HP1, irradiated with 3 Gy IR and 48 h later co-stained with BRCA1 (green) and 53BP1 (red). The fluorescence intensity was quantified along a line drawn through the centre of 53BP1 foci to provide a fluorescence intensity profile using the softWoRx^®^ suite software. In control cells 53BP1 has a bimodal distribution with BRCA1 being localized within the core of diminished intensity. (**C**, **D** and **E**) Similar quantification was carried out following siRNAs targeting control (CTR), SETDB1, SUV39 and HP1. The results show a monomodal distribution of 53BP1. The width of the peak for 53BP1 and BRCA1 are smaller than in the siCTR sample and the BRCA1 peak is off set relative to the 53BP1 peak. (**F**) Quantification of the width of 53BP1 foci. The foci width was determined by measuring the distance between the outer edges of the peaks at 50% intensity. (**G**) BRCA1 intensity following siRNA SETDB1, SUV39 or HP1 was reduced compared to control siRNA. In panels A–D, the maximum Y axis value was automatically set to the maximum fluorescence intensity of 53BP1 (left axis) and BRCA1 (right axis) giving the same size peak irrespective of absolute intensity. Note that BRCA1 intensity is about 10-fold less than the 53BP1 intensity. This panel shows the comparative intensity of BRCA1 following siRNA targeting control (CTR), SETDB1, SUV39 or HP1. Asterisks shows statistically significant difference between control and siRNA SETDB1, SUV39 or HP1 (*P* < 0.005; *t*-test).

To quantify these changes within the foci, we used our previously described procedure where fluorescence intensity profile images were quantified along a line drawn through the centre of the foci at 8 h post IR (26) (Figure 5A). Consistent with previous findings, we observed that at 0.5 h post IR, 53BP1 has a monomodal distribution but by 8 h a bimodal distribution is observed with a core of reduced 53BP1 intensity (Figure 5A; 0.5 h images are not shown but see (26)). This is accompanied by an overall increase in width of the 53BP1 foci demonstrating the repositioning of 53BP1 to the periphery of enlarged foci. Of note, we have previously observed that the enlargement and creation of a devoid core represent distinct steps since siRNA POH1 (a component of the proteasome) allows expansion of 53BP1 but the devoid core does not form (26). We note also that quantitative analysis of 53BP1 intensity demonstrates a core of reduced intensity (Figure 5B). However, analysis of individual deconvolved images demonstrate a more marked 53BP1-devoid core. We have, therefore used this terminology in the ensuing discussion, although formally we cannot distinguish between a devoid core versus a core of reduced intensity. Strikingly, following siRNA HP1, SUV39 or SETDB1 we failed to observe the repositioning of 53BP1 and, importantly there was no evidence for the formation of a 53BP1 devoid core (Figure 5C–E). We also assessed the position of BRCA1 using the same procedure by aligning the centre of the BRCA1 peak relative to the 53BP1 peak. This analysis revealed that, whereas in control cells the BRCA1 peak lies between the two 53BP1 peaks, following depletion of HP1, SUV39 or SETDB1, the peak of BRCA1 intensity was offset relative to the 53BP1 peak. Further, the width of the 53BP1 foci at 8 h post IR following siRNA HP1, SUV39 or SETDB1 was reduced relative to that in control cells (Figure 5F). Although less marked, the width of BRCA1 foci was also reduced, consistent with the findings above that BRCA1 foci do not enlarge by 8 h following depletion of the compacting factors as they do in control cells. Although analysis of individual images of BRCA1 in control cells suggested a core of reduced intensity (Figure 4C), this was not evident from the intensity distribution analysis (Figure 5B), most likely because the latter procedure represents the average of multiple foci and is, therefore, less sensitive in detecting a reduced core. In these images, the fluorescence was set to an arbitrary level to give a similar size peak of BRCA1 and 53BP1 to assess distribution. We also estimated the intensity of BRCA1 at foci following depletion of the compacting factors relative to that in control cells, to enable a comparison of levels. This analysis demonstrated the reduced intensity of BRCA1 at IRIF following siRNA of the compacting factors, a feature that was evident throughout the analysis and that likely contributes to the reduced detection of BRCA1 foci (Figure 5G).

### SETDB1 is recruited to DNA damage sites

**Figure 6.**
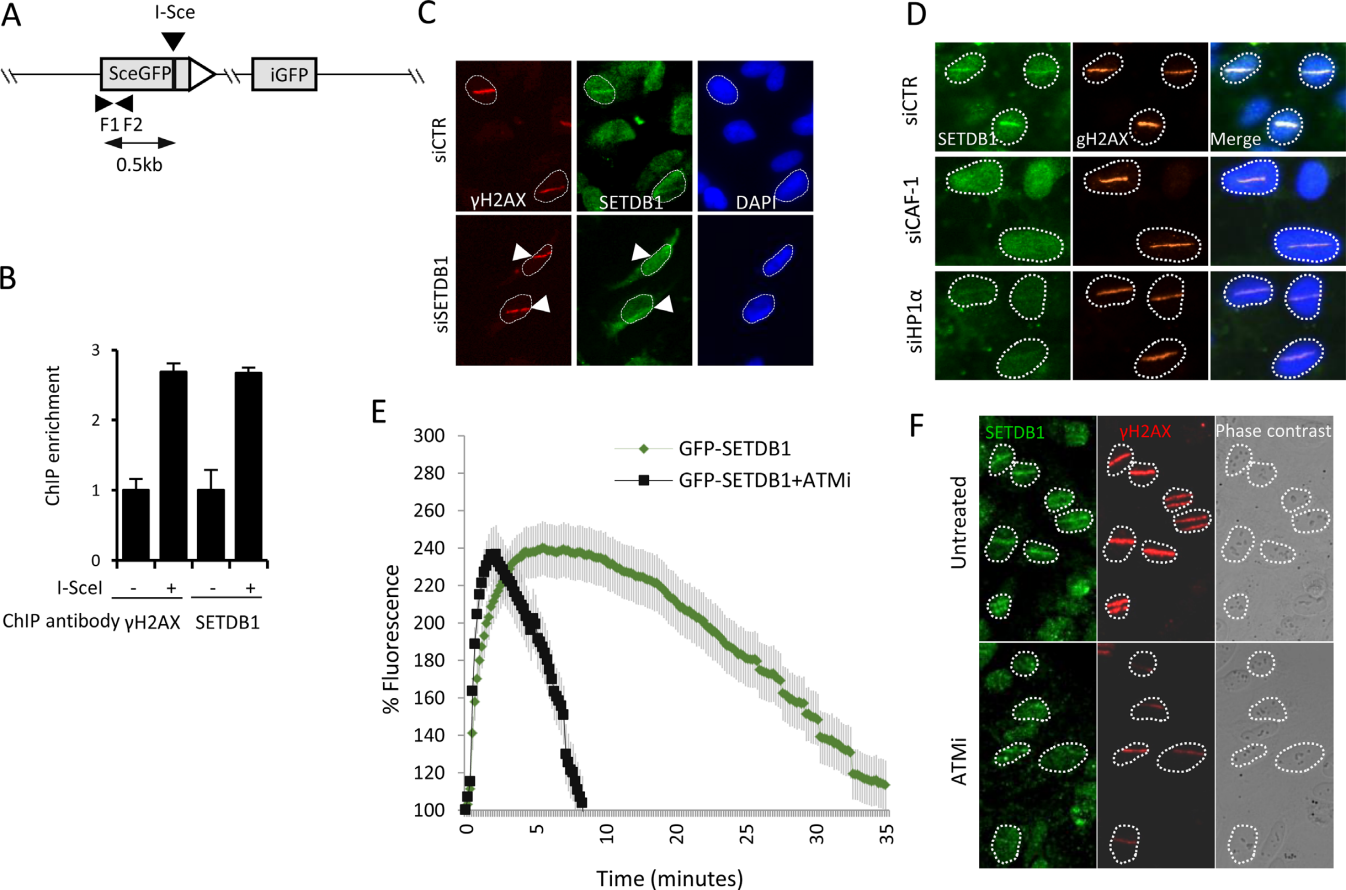
SETDB1 is recruited to DSB sites. (**A**) The pCBA*Sce1* plasmid used to assess protein localization. F1 and F2 represent a primer pair used in panel **B**. The primers are located 500 bp upstream of the I-*SceI* recognition site. (B) HeLa DR-GFP cells were transfected with or without pCBASceI plasmid and processed for the ChIP assay using α—yH2AX and α-SETDB1 antibodies at 24 h post-transfection. DNA was recovered from immunoprecipitates and quantified by real time quantitative PCR. (**C**) U2OS cells were transfected with siRNA targeting control (CTR) or SETDB1. Cells were pre-photosensitized with 10 pM BrdU for 48 h and irradiated with a 405 nm laser. Twenty minutes post-micro irradiation, cells were fixed after pre-extraction and stained for γH2AX, SETDB1 and DAPI. (**D**) U2OS cells were transfected with siRNAs targeting control (CTR), p150CAF-1 (CAF-1) or HP1α and 48 h later, cells were microirradiated as in C. Twenty minutes later, cells were pre-extracted, fixed and stained for SETDB1, γH2AXor DAPI. (**E**) U2OS cells transfected with the pEGFP-SETDB1. After 48 h transfection cells were irradiated with a 405 nm laser. A total of 10 μM ATM inhibitor (KU-55933) was added to the cells 1 h prior to microirradiation. The GFP signal intensity was quantified along the laser track in untreated and ATMi-treated cells expressing pEGFP-SETDB1. GFP-signal intensity was monitored in 40 cells for 35 min post IR. Results represent the mean ± S.E.M of three experiments. (**F**) U2OS cells were treated with 10 μM ATM inhibitor (KU-55933) for 1 h prior to microirradiation as in panel C. Twenty minutes later, cells were processed for immunofluorescence using α-SETBD1 or α—yH2AX antibodies. Reduced γ-H2AX formation and SETDB1 accumulation was observed in cells treated with ATMi compared to control cells.

In addition to being required for HR, previous studies have reported that HP1α and SUV39 are recruited to DSB sites (15,17,19). Since SETDB1 represents a novel component required for HR, we examined whether, like the other compacting factors, it is also recruited to the sites of DNA damage. Due to limitations of the available SETDB1 antibodies and a level of chromatin bound SETDB1 without DNA damage, we were unable to reliably detect SETDB1 recruitment to γH2AX foci. As an alternative procedure to monitor a role for SETDB1 in the DDR, we used a ChIP-based assay following introduction of a site-specific DSB (Figure 6A). As expected, following I-*Sce1* expression, chromatin bound γH2AX increased. Strikingly, we observed a similar increase in chromatin bound SETDB1 (Figure 6B). Whilst this does not verify that SETDB1 is specifically recruited to DNA damage sites, it provides initial evidence that the level of chromatin bound SETDB1 increases after DNA damage.

Next, we examined the recruitment of SETDB1 to the sites of DNA damage induced by 405 nm laser irradiation in photosensitized cells. First, we examined the recruitment using α-SETDB1 antibodies at 20 min post laser irradiation. At this time point, SETDB1 recruitment to laser tracks could be readily observed (Figure 6C). Recruitment was observed in all cells with laser-induced damage assessed by γH2AX and was not specific to G2 cells (data not shown). Since HP1α and p150CAF-1 are also recruited to 405 nm laser-induced DNA damage (15,17), we examined whether SETDB1 recruitment is dependent upon HP1 and p150CAF-1. We observed substantially diminished recruitment of SETDB1 following siRNA HP1α or p150CAF-1 (Figure 6D) suggesting that efficient SETDB1 recruitment requires HP1α and p150CAF-1. Finally, previous work suggested that HP1α is only transiently retained at laser tracks. To examine the kinetics of SETDB1 retention, we used live cell imaging to monitor the retention of EGFP-SETDB1 following its expression in U20S cells. Significantly, we observed optimal recruitment of SETDB1 between 5–10 min post treatment, with a gradual decrease over the following 30 min (Figure 6E), kinetics which appear similar to that reported for HP1 (15). At 20 min post laser treatment, substantial SETDB1 is still retained, consistent with our findings above, although the recruitment peak has been passed. Collectively, these findings show that SETDB1 is recruited to the sites of DNA damage via a process that, at least to some degree, requires HP1α.

Finally, to gain evidence that SETDB1 might be a further component of the DDR, we examined whether ATM was required for its recruitment. In our approach using α-SETDB1 antibodies at 20 min post laser treatment, we observed that SETDB1 recruitment to laser tracks is substantially diminished in the presence of an ATM inhibitor, KU55933 (Figure 6F). As reported previously, the formation of γH2AX is reduced in the presence of the ATM inhibitor, consistent with the notion that DNA-PK can phosphorylate H2AX when ATM is inhibited but does so less efficiently than ATM (32). The recruitment of SETDB1 was almost abolished, however. To examine, the kinetics of SETDB1 recruitment in the presence of ATMi, we carried out live cell imaging as described above and observed, surprisingly, that SETDB1 is initially recruited normally to laser tracks but is rapidly lost within 10 min (Figure 6E).

### Depletion of HP1, SUV39 or SETDB1 impairs sister chromatid cohesion

**Figure 7.**
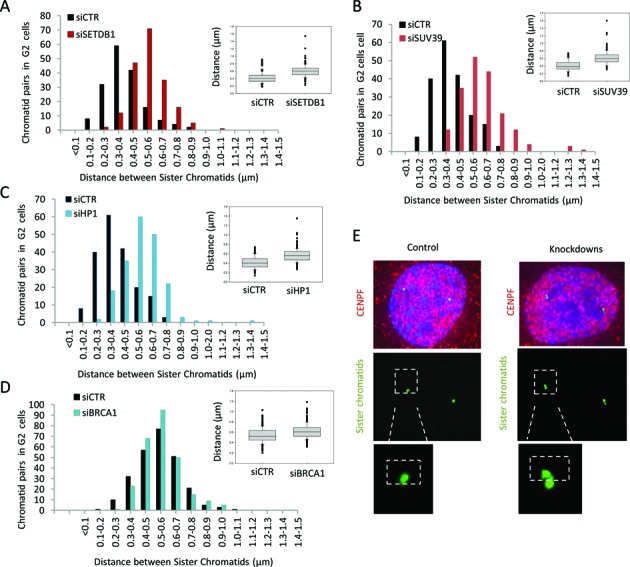
Depletion of HP1, SUV39 or SETDB1 impairs sister chromatid association. (**A, B, C** and **D**) The distance between sister chromatids was quantified in 1BR3 hTERT G2 cells transfected with siRNAs targeting control (CTR), SETDB1 (A), SUV39 (B), HP1 (C) and BRCA1 (D) using IF-FISH assay. The SoftWoRx^®^ suite software was used to measure distance between the sister chromatids. The distance between the outer edges of the foci were measured. The distances between signals were measured from three independent experiments and the distribution was plotted as a histogram. Each individual experiment gave a similar difference in distribution. The box plot represents the corresponding clustered columns. Distribution counts from the FISH experiments were analysed using Mann–Whitney Rank Sum Test. (**E**) IF-FISH images for control siRNA and siRNA SETDB1. Not that since then two FISH spots frequently overlap, the distance between the outer edges were measured. The distances do not, therefore, represent actual sister separation differences but provide a readout for sister association.

The findings above suggest that the compacting factors function in a step of HR downstream of the initiation of resection. A critical step during HR is engagement of the damaged strand with the sister chromatid. Whilst the sister chromatid may be readily available during the events proceeding replication, they may be more distant in G2 phase cells. We considered whether chromatin compaction might enhance sister association in G2 phase cells. To assess this, we examined the distance between sister chromatids in control cells and following siRNA of HP1, SUV39 or SETDB1. To assess sister chromatid association, we used a probe located in the centromeric region of chromosome 10 for FISH analysis and quantified the distance between the two foci in G2 phase cells (33). Strikingly, we observed enhanced separation of the centromeric probes following siRNA of HP1, SUV39 or SETDB1 (Figure 7A–C).

BRCA1 has also been reported to function in the formation of pericentric heterochromatin (34). Thus, we also assessed the impact of siRNA BRCA1 on sister chromatin association using the same procedure. Although we consistently observed a small increase in sister separation following siRNA BRCA1 (see Figure 7D), this difference varied between experiments and was clearly substantially smaller than observed following siRNA of the compacting proteins.

## DISCUSSION

Previous studies have shown that the progression of HR requires processes that enhance chromatin decompaction as well as the recruitment of factors required for chromatin repression (19). Whilst there may be temporal differences in the requirement for compaction versus relaxation, the changes may also be distinct at specific locations relative to the DSB site. The requirement for chromatin modifications such as ubiquitylation has been well analysed (35). However, although recent studies have provided evidence that factors, including HP1, SUV39H1 and KAP1, that are required for chromatin compaction play an essential role in HR, a dissection of their precise function has not been undertaken (15,19). Here, we report that SETDB1 is a further compacting factor required for HR. Like siRNA HP1α/β or SUV39, siRNA SETDB1 results in a diminished level of H3K9me3. SETDB1 recruitment and retention at laser tracks occurs with similar kinetics to that of HP1α and SUV39H1 (15,19) and, like HP1, is dependent upon p150CAF-1. However, surprisingly, whilst the retention of SUV39 at laser tracks is enhanced following ATM inhibition, SETDB1 is prematurely released when ATM is inhibited (19). SUV39's release from damage sites has been proposed to depend upon the phosphorylation of KAP1 by ATM. The basis for the distinction with SETDB1 is currently unclear and requires further investigation. However, our focus in this study lies on the role of the compacting proteins during HR. To this end, we provide evidence that their depletion confers a remarkably similar phenotype suggesting that they function in a similar process, which we propose necessitates chromatin repression. Whilst these factors are all required for heterochromatin formation, we do not distinguish here whether they simply enhance chromatin compaction at DSB sites versus generating a heterochromatic microenvironment.

Previous studies have shown that in G2 phase cells, the progression of resection involves an initiation step involving CtIP/MRN (4,5). If this process is prevented by siRNA CtIP, then, although HR cannot take place, DSB repair can ensue by NHEJ suggesting that this initiation step represents an event committing to HR (2,3). Our findings reveal that the compacting factors are dispensable for the initiation of resection. The progression of resection and efficient loading of RAD51 necessitates multiple changes at damage induced foci, however. BRCA1 is essential for this step of HR and functions to relieve the inhibitory impact of 53BP1 (8,9). The compacting factors appear to be required during this step of HR. Intriguingly, studies have proposed that RIF1 is the final effector that inhibits resection via its interaction with 53BP1 (10–12). However, loss of the compacting factors does not result in enhanced levels of RIF1 foci in G2 phase cells as observed following depletion of BRCA1, showing that RIF1 is not the sole factor impeding the extension step of HR. This raises the possibility that 53BP1 can be inhibitory to resection in a RIF1-independent manner.

Our findings strongly suggest that the compacting factors are not essential for BRCA1 recruitment to DSBs but they impede its correct localization relative to 53BP1 and its function in promoting the repositioning of 53BP1 as HR ensues. Since BRCA1 intensity at foci increases as HR ensues due to its enlargement and repositioning, the BRCA1 foci appear reduced in size and numbers at 8 h in G2 cells following loss of the compacting factors. This is consistent with, but extends, previous studies showing that BRCA1 foci are reduced in number following siRNA HP1. Since a reduction in foci size could readily explain the observed decrease in foci numbers due to scoring inaccuracies, we propose that the primary impact of loss of compacting factors is a failure to promote the repositioning of BRCA1 as HR progresses.

The progression of HR must involve engagement of the damaged strand with the undamaged template. We considered that the failure to progress resection and Rad51 assimilation following depletion of the compacting factors could potentially reflect a failure to engage with the sister homologue. This hypothesis was in part prompted by the finding that 53BP1 foci undergo a two-fold enlargement during HR, which we considered could represent its transfer onto the undamaged strand (36). This two-fold enlargement does not occur in G1 phase cells. Given this possibility, we evaluated whether chromatin compaction might influence the association between sister chromatids. Strikingly, we found that in undamaged cells, depletion of any of the three compacting factors caused an increase in the separation between sister chromatids. Moreover, the magnitude of this effect generally paralleled the impact of these factors in our HR assays (siRNA HP1 and SUV39 causing a greater impact than siRNA SETDB1), and most notable in diminishing BRCA1 intensity at foci, raising the possibility of a causal relationship.

Based on these findings, we propose the following model. Following initiation, resection progresses in a 5′ to 3′ direction followed by loading of RAD51. This promotes assimilation of the damaged strand onto the undamaged strand, which is required for further extension of resection, further loading of RAD51 and the completion of HR. These latter steps also require chromatin changes on the undamaged template, including the recruitment of 53BP1. BRCA1 triggers this process, which requires new ubiquitylation events and proteasome-mediated degradation (26). Importantly, this step also necessitates the ability to engage with the sister homologue. We propose that it is this step that is promoted by the transient recruitment of chromatin repressive factors. This is consistent with our previous suggestion that the enlargement of 53BP1 represents its association on the undamaged strand, which is likely to require appropriate sister chromatin cohesion (26,36). Currently, we have only shown that diminished chromatin compaction as evidenced by reduced H3K9me3 levels causes enhanced sister separation at centromeric regions, where compacting factors are high. Given that the repressive factors are recruited to DSB sites, we propose that they may serve to transiently enhance sister association at such sites. Although currently only correlative, verification of this role necessitates approaches to allow sister chromatid cohesion at DSBs to be monitored.

In summary, we demonstrate that SETDB1 is a further compacting factor required for HR and that siRNA of SETDB1 confers an HR defective phenotype identical to that conferred by depletion of HP1 or SUV39, which also reflect the phenotype conferred by loss of BRCA1. We define the stage during HR when the compacting factors function showing that they are dispensable for the initiation of resection but are required to promote enlargement of 53BP1 foci, which is itself required for the progression of resection. They are dispensable for BRCA1 recruitment but impede its enlargement and correction positioning within foci. Finally, we demonstrate that a distinct impact of depletion of these compacting factors is to enhance the separation of sister chromatids at centromeric regions. Based on this finding, we propose a model for how the compacting factors may promote sister chromatid engagement during HR.

## Supplementary Material

SUPPLEMENTARY DATA
