# Supplementary material for: SETDB1, HP1 and SUV39 promote repositioning of 53BP1 to extend resection during homologous recombination in G2 cells
Source: Nucleic Acids Res. 2015 Jul 22;43(16):7931–44. doi: 10.1093/nar/gkv722 (PMC4652757; doi:10.1093/nar/gkv722)
Supplement: SUPPLEMENTARY DATA [file supp_43_16_7931__index.html]

SETDB1, HP1 and SUV39 promote repositioning of 53BP1 to extend resection during homologous recombination in G2 cells — SETDB1, HP1 and SUV39 promote repositioning of 53BP1 to extend resection during homologous recombination in G2 cells — SETDB1, HP1 and SUV39 promote repositioning of 53BP1 to extend resection during homologous recombination in G2 cells — SUPPLEMENTARY DATA 

# SETDB1, HP1 and SUV39 promote repositioning of 53BP1 to extend resection during homologous recombination in G2 cells

## SUPPLEMENTARY DATA

- SUPPLEMENTARY DATA
