# Supplementary material for: SETDB1, HP1 and SUV39 promote repositioning of 53BP1 to extend resection during homologous recombination in G2 cells
Source: Nucleic Acids Res. 2015 Jul 22;43(16):7931–44. doi: 10.1093/nar/gkv722 (PMC4652757; doi:10.1093/nar/gkv722)

Supplementary Figure 1.

A. The percentage of G2 (CENPF+) cells was determined after knockdown of SETDB1, SUV39 or HP1. The ratio of $CENP^{+}$: DAPI cells was assessed in a minimum of 200 cells.

B and C. Following siRNA mediated knockdown of control (CTR), SETDB1, SUV39, HP1, 1BR3 hTERT cells were irradiated with 3 Gy, RPA (C) and RAD51 (D) foci number were enumerated at the indicated times.

Supplementary Figure 2.

A. Following transfection of U2OS DR-GFP cells with siRNAs targeting control (CTR), SETDB1, SUV39, HP1 or BRCA1, cells were re-transfected with pCBA*SceI* and stained with propidium iodide. The percentage of G1, S and G2/M phase cells was assessed by FACS analysis.

B. The *I-SceI* plasmid (pCBA*SceI*) includes an HA-Tag. After transfection of U2OS DR-GFP cells with pCBA*SceI*, *I-SceI* expression level were determined by staining with HA antibody**.**

C. HR efficiency was also determined after SETDB1 knockdown using each siRNA oligonucleotide. Results represent the mean ± S.E.M of three experiments. Asterisks indicate a statistically significant difference between siCTR and siSETDB1#A or siSETDB1#B ($P < 0.05$; t-test).

D. Knockdown efficiency of two distinct siRNA SETDB1 oligonucleotides, singly or combined, was assessed by western blotting.

E and F. Fallowing transfection of siRNAs targeting control (CTR), SETDB1, SUV39 or HP1, H1299 dA3-1 cells, which harbour a construct that allows NHEJ to be monitored, were re-transfected with pCBA*SceI* (3,30). The percentage of GFP^{+} cells was measured by FACS 48 h after transfection with *I-Sce1*. GFP^{+} cells were normalised to the percentage of EGFP positive cells and the level of the S/G2 population (**E**).

The level of HA-tagged *I- SceI* was assessed by staining with HA antibody following knock down (**F**).

Supplementary Figure 3.

A. 1BR3 hTERT cells were transfected with siRNAs targeting control (CTR) or EXO1/BLM. The knockdown efficiency was verified by immunoblotting.

B and C. 1BR3 hTERT cells were transfected with siRNAs targeting control (CTR) or SETDB1, 48 h later cells were irradiated with 3 Gy and co-stained with γH2AX/ 53BP1 or γH2AX/BRCA1 antibody at the indicated times post IR. The percentage of overlapped γH2AX/ 53BP1 foci (**A**) and γH2AX/BRCA1 foci (**B**) was quantified in deconvolved Delta Vision 2D images by the softWoRx® suite software. The Pearson Coefficient of Correlation monitors how closely two intensities overlap on a pixel-by-pixel basis (full overlap is 1.0).

Supplementary figure 1

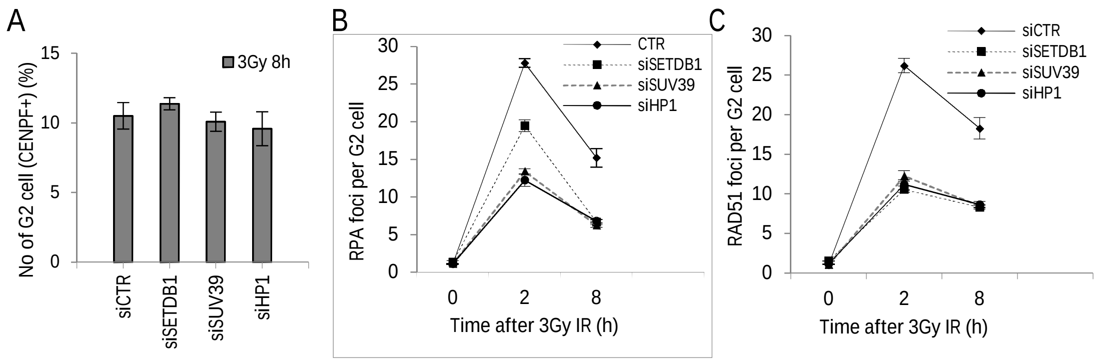

Supplementary figure 2

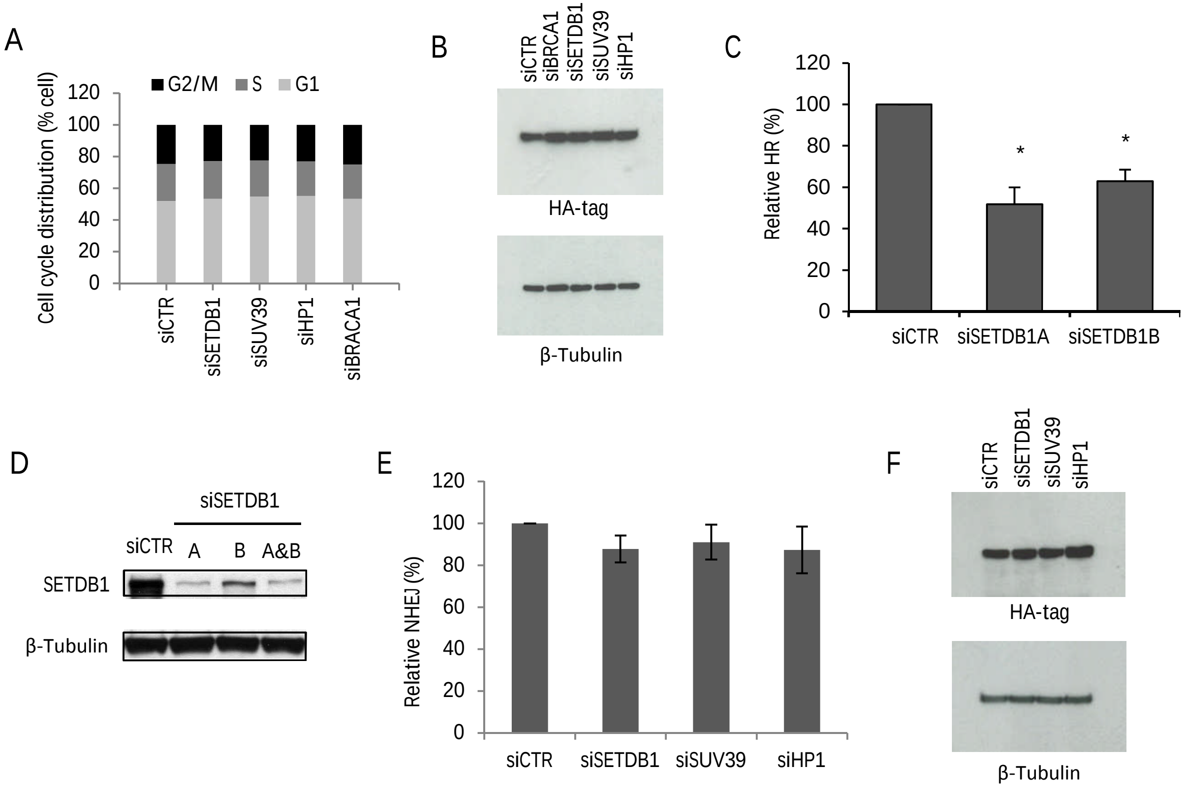

Supplementary figure 3

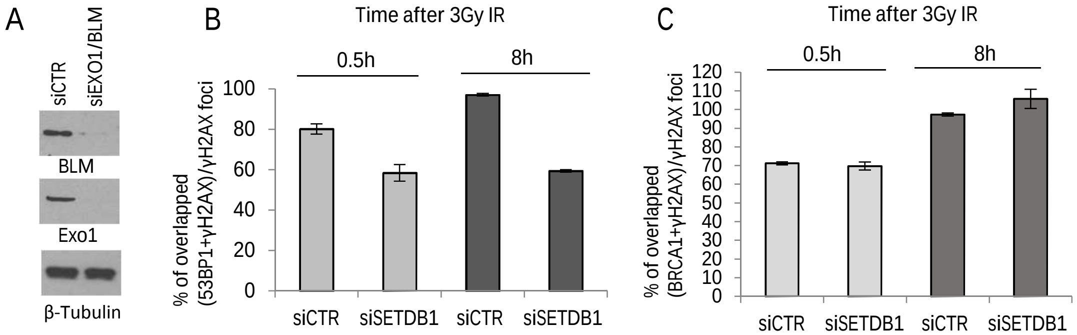

Supplement: SUPPLEMENTARY DATA [file supp_gkv722_nar-00330-d-2015-File009.pdf]
